# DQN-Based Adaptive Intersection Control for Warehouse AGVs in SUMO with a Proposed VLC Architecture †

**DOI:** 10.3390/s26196212

**Published:** 2026-09-30

**Authors:** Paula Louro, Gonçalo Galvão, Susana Amaral, Manuela Vieira, Manuel A. Vieira

**Affiliations:** 1 Instituto Superior de Engenharia de Lisboa (ISEL), Universidade Politécnica de Lisboa, R. Conselheiro Emídio Navarro, 1959-007 Lisboa, Portugal; goncalo.galvao@isel.pt (G.G.); a33191@alunos.isel.pt (S.A.); manuela.vieira@isel.pt (M.V.); manuel@deetc.isel.ipl.pt (M.A.V.); 2 Instituto de Desenvolvimento de Novas Tecnologias-Center of Technology and Systems-Laboratório Associado de Sistemas Inteligentes (UNINOVA–CTS-LASI), Universidade NOVA de Lisboa, Quinta da Torre, Monte da Caparica, 2829-516 Caparica, Portugal; 3 NOVA School of Science and Technology, Universidade NOVA de Lisboa, Quinta da Torre, Monte da Caparica, 2829-516 Caparica, Portugal

**Keywords:** Automated Guided Vehicles (AGVs), Deep Q-Network (DQN), Multi-Agent Reinforcement Learning (MARL), Simulation of Urban Mobility (SUMO), adaptive intersection control, warehouse traffic management, Visible Light Communication (VLC)

## Abstract

Congestion at shared intersections can limit the scalability of Automated Guided Vehicle (AGV) fleets in high-density warehouses. This study evaluates a Deep Q-Network (DQN) controller for adaptive intersection management in a SUMO-based warehouse model with five crossings, including the shared central crossing CP1. After one 200-episode training run, the DQN was evaluated in three seeded 6200 s simulations per demand scenario. Against a study-specific cyclic fixed-time controller at 250, 350, and 400 AGVs/h, the DQN achieved unweighted mean reductions of 62.9% in signal queue, 59.1% in average waiting time, and 18.0% in AGVs waiting to load, while loading/unloading occupancy changed by less than 1%. A matched 750 AGVs/h stress test showed that the benefit did not persist after saturation: at CP1, the mean signal queue increased from 13.15 to 30.68 AGVs and the mean number waiting to load from 9.64 to 15.93 AGVs. Sustained CP1 congestion began between 350 and 400 AGVs/h for the tested layout. Retained test outputs quantify test-level dispersion, but the single training run cannot estimate training variability. These results quantify the DQN traffic-control policy under ideal availability of SUMO state information and do not validate a VLC system. VLC is presented separately as a proposed future implementation layer; communication delay, packet loss, occlusion, and other physical-channel effects were not modelled.

## 1. Introduction

The ongoing digital transformation of industrial production and logistics has accelerated the deployment of Automated Guided Vehicles (AGVs), which are central to flexible intralogistics and autonomous material transport [1,2,3,4,5,6]. AGVs are driverless transport units that move materials and goods along controlled routes in warehouses, factories, and distribution centres. Their evolution from simple guided carts to sensor-rich autonomous mobile systems has enabled more adaptive navigation, fleet coordination, and production support. Across manufacturing and logistics, these systems can improve operational efficiency, reduce handling damage, and enhance safety when their navigation and traffic-control functions are appropriately coordinated.

AGVs are primarily designed for the horizontal transport of materials, performing supply and disposal tasks between production and storage areas, supporting assembly operations, and handling goods retrieval and distribution [6]. More recent systems also operate in specialised environments and may incorporate robotic manipulators or collaborative functions. These capabilities position AGVs as components of Industry 4.0 infrastructures, in which data-driven automation and cyber–physical integration support operational flexibility and resilience.

The operation of an AGV relies on the integration of three fundamental subsystems: navigation, actuation and control, and communication. Navigation determines vehicle position and trajectory, which can be achieved using line tracking, inductive or magnetic guidance, optical sensors, or laser reflectors [7]. Advanced AGVs increasingly rely on simultaneous localisation and mapping (SLAM) algorithms, which integrate data from multiple sensors, including LiDAR, cameras, ultrasonic devices, and inertial measurement units, to construct and update real-time maps of the operational environment [8]. The actuation and control subsystem manages motion planning, drive systems, and load-handling mechanisms, while the communication subsystem enables real-time interaction between AGVs and fleet management platforms, ensuring coordinated and safe operation within dynamic industrial environments.

As AGV deployment scales up, efficient coordination becomes increasingly critical. High-density operation within warehouses, where multiple vehicles share narrow aisles, intersections, and loading zones, often leads to congestion, queue formation, and reduced throughput if traditional traffic management strategies are employed. To address these challenges, AGV fleet coordination is increasingly treated as a dynamic traffic management problem, where vehicles act as autonomous agents navigating within an indoor transportation network. Each agent adapts its movement based on local traffic conditions and prioritisation policies, enabling adaptive scheduling and congestion mitigation.

The primary contribution evaluated in this study is the DQN-based intersection-control policy; VLC is considered separately as a proposed future communication and localisation architecture. Visible Light Communication (VLC) uses intensity-modulated light-emitting diodes for illumination and data transmission and has been widely investigated for indoor positioning and vehicular communication [9,10,11,12,13,14,15]. Optical signals can be detected by photodiode receivers, including multilayer a-SiC:H devices developed for wavelength-selective optical communication [16]. Compared with radio-frequency-only solutions, VLC offers licence-free optical spectrum, immunity to electromagnetic interference, spatial confinement, and compatibility with existing LED lighting. These properties motivate its consideration for future AGV localisation and traffic-state exchange, although the optical link is not simulated in the present evaluation.

As a proposed future implementation layer, the architecture uses ceiling luminaires, wall-mounted signalling units, and AGV headlamps as optical transmitters, while on-board photodiodes provide reception. The resulting topology supports lamp-to-vehicle (L2V), vehicle-to-vehicle (V2V), vehicle-to-infrastructure (V2I), and infrastructure-to-vehicle (I2V) links, consistent with reported white-LED transmission, measured vehicular VLC channels, and V2I channel studies [17,18,19]. L2V is intended primarily for positioning and navigation, whereas V2V, V2I, and I2V support traffic-state and control-information exchange. This architecture is not part of the evaluated SUMO 1.19 control loop.

The empirical focus of this study is adaptive intersection control evaluated using the Simulation of Urban Mobility (SUMO) platform, adapted to represent AGVs travelling along predefined warehouse aisles and through controlled intersections [20,21]. SUMO provides vehicle-level states and traffic metrics, including position, speed, lane occupancy, queue length, and waiting time. The adaptive controller uses these states to select intersection signal phases; its reward favours traffic progression and penalises waiting and queue formation, following established reinforcement-learning approaches to traffic-signal control and state representation [22,23,24]. The simulation therefore evaluates intersection coordination rather than AGV route planning or task reallocation.

The evaluated system is the DQN traffic controller coupled to SUMO; VLC is retained only as a proposed communication and positioning layer for future deployment. In the present simulations, however, SUMO supplies the required state variables directly to the control layer, and message delivery is assumed to be available without packet errors, communication delay, line-of-sight blockage, or handover interruption. The reported performance therefore quantifies the traffic controller under idealised communication availability; it does not constitute a physical-layer validation of bit-error rate, latency, resistance to ambient-light interference, occlusion resilience, or RF fallback. The multilayer photodetector and hybrid VLC/RF elements are consequently treated as design provisions supported by prior device research [25] and as subjects for future experimental evaluation.

Deep reinforcement learning has become an established approach to adaptive traffic-signal control under variable demand [26,27,28]. Cooperative multi-agent formulations address interactions among neighbouring controllers, including Q-value transfer for multi-intersection coordination [29] and broader cooperative MARL architectures [30]. Related surveys also show how deep reinforcement learning can support communication and networking decisions [31]. In parallel, vehicular VLC studies have examined V2V reliability and capacity [32], while CTDE architectures have been investigated for scalable multi-agent pathfinding [33]. In warehouse environments, MARL has been applied to cooperative multi-AGV control [34], energy-efficient routing [35], dynamic scheduling [36], and safety-policy generation under uncertainty [37]. Rule-based adaptive traffic control supported by cooperative VLC provides a relevant conceptual antecedent [38], but the present study instead evaluates a DQN in which phase selection and duration respond jointly to local occupancy, speed, queues, and downstream conditions. The practical integration of this controller with VLC-based information exchange remains a separate implementation question.

The conference paper “Visible Light Communication for Adaptive AGV Fleet Coordination” examined a queue-based controller in which the traffic phases followed a predefined cyclic sequence and their durations were adjusted from the traffic conditions observed on the incoming lanes of each crossing point [39]. The present work builds on that rule-based strategy by using a DQN to select which of the eight vehicular phases should be activated from the current local occupancy and speed state. The controller is therefore not restricted to the predefined cyclic order: both the activation sequence and the effective service duration can adapt, with consecutive selections of the same phase extending its green time in 8 s increments. The earlier work demonstrated queue-responsive timing within a cyclic VLC-supported framework, whereas the present study evaluates learning-based phase selection in SUMO. Conversely, the current simulations assume ideal availability of traffic-state information and do not model the VLC physical or network layer; they therefore neither reproduce nor further validate the optical-link results reported in the earlier study.

A preliminary version of the warehouse scenario and its VLC-enabled control concept was presented in an earlier conference paper [40]. That study reported initial controller time series for 250, 500, and 750 AGVs/h and identified the need to refine the CP1 operating boundary using intermediate demand levels. The present article substantially extends that work by (i) specifying the adapted DQN architecture, 160-variable observation, vehicle-only reward, and P1–P8 action mapping; (ii) adding the 350, 400, and 450 AGVs/h transition analysis; (iii) introducing matched fixed-time comparisons at 250, 350, and 400 AGVs/h and a separate 750 AGVs/h stress test; and (iv) separating the evaluated SUMO controller from the proposed VLC implementation layer and reporting the resulting reproducibility limitations.

Against this background, the control-focused contributions of this study are as follows:

- SUMO-based warehouse evaluation model: The controller is coupled to an adapted warehouse network with five crossing points, predefined aisles, loading or unloading activity, and CP1 shared by the horizontal and vertical corridors.
- Comparative fixed-time benchmark: The DQN controller is compared with a study-specific cyclic fixed-time policy in matched 250, 350, and 400 AGVs/h scenarios, with a separate 750 AGVs/h stress comparison. This benchmark, which was not included in the preliminary conference paper, uses queue length, waiting time, service-queue, and traffic-flow indicators.
- Layout-specific operating-regime analysis: Following the transition-region question raised in the preliminary study [40], the present work adds 350, 400, and 450 AGVs/h scenarios and places the onset of sustained CP1 congestion between 350 and 400 AGVs/h for the reported layout, service process, and run.
- Proposed VLC implementation architecture: L2V, V2V, V2I, and I2V links are mapped to positioning and traffic-state exchange as a future deployment concept. The VLC layer is not coupled to the experiments and is not presented as the cause of the observed controller gains.

The remainder of this paper is organised as follows. Section 2 defines the evaluation design, documents the proposed VLC implementation architecture, describes the adapted SUMO warehouse model, and specifies the DQN controller and comparator. Section 3 reports training behaviour and traffic-performance results, including the fixed-time benchmark, the operating-regime analysis at CP1, and a separate 750 AGVs/h stress test. Section 4 summarises the findings, limitations, and priorities for controller reproducibility and future communication-layer validation.

## 2. Methodology

The evaluated system is a combined DQN intersection controller coupled to an adapted SUMO warehouse model. Five agents regulate the crossing phases, with the DQN selecting a signalling phase from the local traffic state. The controller uses eight vehicular phases, a 160-variable state, and an AGV-only reward. A study-specific cyclic fixed-time policy provides the comparison condition. The VLC hardware and protocol described in Section 2.1 constitute a proposed future implementation layer only; no optical channel is coupled to the traffic experiments, which assume ideal availability of SUMO state variables.

AGVs operate on the fixed warehouse layout described in Section Layout of the Indoor Environment and AGV Movement under network-wide arrival-rate scenarios ranging from 250 to 750 AGVs/h. SUMO version 1.19 is used. Vehicle arrivals follow a Weibull distribution. The stated arrival rates represent the total number of AGVs entering the complete network per hour, rather than a rate assigned independently to each entry or corridor. Approximately 65% of the total flow is assigned to the stronger CP0–CP1–CP2 axis and 35% to CP3–CP1–CP4; across both axes, approximately 75% of AGVs proceed straight, and 25% turn left. Loading and unloading events are configured in SUMO, which records vehicle position, speed, lane occupancy, queue formation, waiting time, and service activity. Only one layout is evaluated. The detailed origin–destination and task-assignment rules are not retained in the available materials.

At each simulation step, the traffic-state variables generated by SUMO are mapped to the information fields of the proposed VLC links and made available to the intersection controller. This is a logical emulation of information exchange: optical waveforms, propagation loss, packet errors, latency, occlusion, and receiver saturation are not coupled to the SUMO control loop. Consequently, the controller is evaluated under idealised message availability.

The warehouse implementation retains the centralised-training/decentralised-execution architecture of the published urban controller [38]. Five intersection agents contribute transitions to a common replay memory and share one online DQN and its target network during training; during execution, each agent selects a signalling phase from its local intersection state. The implemented control scope is limited to intersection phase selection and timing: no independent predictive model, AGV rerouting, dynamic task reallocation, or learned task scheduling is evaluated.

SUMO advances with a simulation step of 1 s, so vehicle dynamics and traffic-state variables are updated once per second. The closed-loop controller uses a variable decision interval equal to the duration of the applied signalling phase. At the end of that phase, the agent at each crossing point observes the updated environment, selects the next action, and calculates the reward attributed to the preceding action. The resulting state–action–reward transitions are stored for training. The precise number and timing of online-network gradient updates within each training cycle were not retained in the available materials; the target-network update is documented separately below.

Controller performance is assessed using the same SUMO 1.19 layout and demand scenarios for two control policies: a cyclic fixed-time comparator and the DQN controller. The DQN was trained once for 200 episodes of 3600 s, with no warm-up period. The network from the final training episode was then evaluated in three 6200 s simulations for each reported demand scenario. The predefined simulation seeds were 10,000, 10,002, and 10,003, and the same seeds were used for the corresponding fixed-time tests, providing matched stochastic demand conditions across controllers. Queue, loading/unloading, and waiting-to-load indicators were sampled once per second, producing 6200 values per crossing point and test; average speed and average waiting time were evaluated minute by minute, producing approximately 103 values per test. The plotted traffic curves and reported scenario values are arithmetic means across the three post-training simulations, and environment-wide queue and service indicators are unweighted arithmetic means of CP0-CP4. The individual test outputs are retained, permitting dispersion across the three post-training simulations to be calculated; this variability does not represent training variability because the DQN was trained only once. Temporal samples within a series are not treated as independent simulation replications. The training-reward trace is the single 200-episode training trajectory and is not an average of three trainings. Because communication is assumed to be ideal, differences between the two conditions quantify the effect of the control policy and cannot be attributed to VLC.

The comparator is a study-specific cyclic fixed-time controller based on the configuration reported in earlier work [38]. It follows the predefined sequence P1–P8. The main phases P1 (north–south) and P5 (west–east), which serve the dominant corridors, receive 40 s of green. The secondary phases P2 (north only), P3 (south only), P4 (north–south left turns), P6 (west only), P7 (east only), and P8 (west–east left turns) receive 20 s of green. Each green is followed by a 4 s yellow interval; no separate all-red interval is applied, and red time is complementary to the active green and yellow intervals. The resulting cycle length is 232 s. The 2:1 green-time allocation was selected as a deterministic operational baseline that gives greater discharge capacity to the more heavily used corridors while periodically serving every movement. These timings are not presented as a universal or industrial warehouse standard, but as a realistic study-specific reference against which the adaptive policy can be evaluated.

### 2.1. Proposed VLC Implementation Architecture for Future Validation

In the simulated scenarios, SUMO generates position, speed, queue length, waiting time, and manoeuvre information for each AGV. These variables populate the logical payloads associated with the L2V, V2V, V2I, and I2V links. The communication architecture therefore defines how information would be exchanged, whereas the mobility-control experiments use the SUMO variables directly and do not model the performance of the optical channel.

#### 2.1.1. VLC Transmitters and Receivers

The proposed VLC transmitter uses tetrachromatic white LEDs with independently addressable red, green, blue, and violet emitters. Four LEDs are arranged in a square, with one selected emitter from each package used for communication while the remaining emitters provide illumination. The nominal peak wavelengths are 620 nm, 530 nm, 470 nm, and 405 nm, with reported linewidths of 24–38 nm, luminous intensities of 340–980 mcd, and a 120° beamwidth. These values define the assumed component configuration; the present study does not report a warehouse-scale transmitter characterisation.

**Figure 1:**
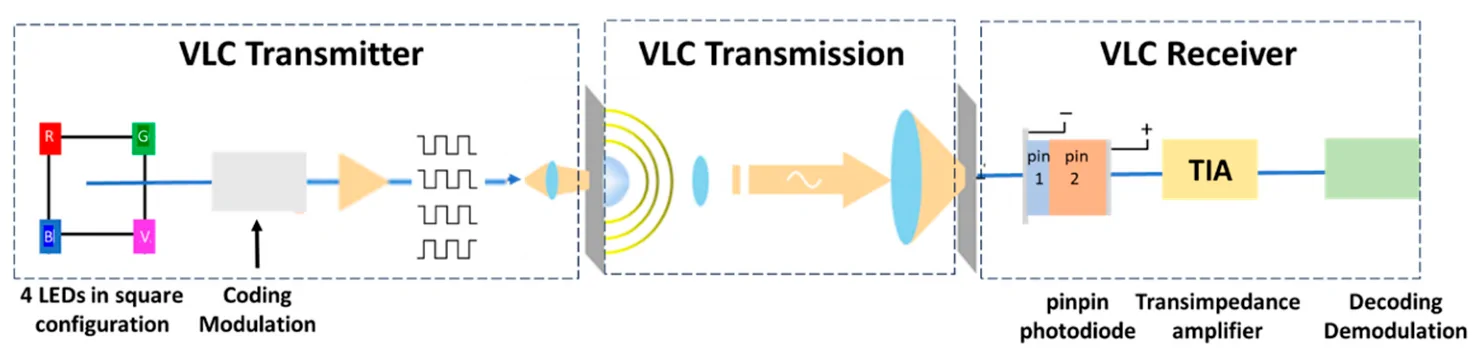
VLC transmitter and receiver.

The receiver concept uses a p-i-n photodetector to collect multiplexed optical signals from up to four emitters. Four binary emitter states yield 2^4^ = 16 possible excitation combinations, which can in principle be mapped to distinct photocurrent levels after calibration. Detection is based on thresholding the measured photocurrent, and data modulation uses on-off keying (OOK). The threshold values, receiver responsivity, signal-to-noise ratio, bit-error rate, and probability of level misclassification are not measured in the present warehouse scenario; therefore, reliable bit recovery is an architectural assumption rather than an evaluated result. The architecture of the VLC system is illustrated in Figure 1.

For completeness, the line-of-sight optical path is described using a Lambertian emission model. The radiant intensity I at irradiance angle φ is expressed as

*I*(*φ*) = *I_N_cos^m^*(*φ*)

where I_N_ is the axial intensity and *m* is the Lambertian order. The simplified channel gain G is written as

$$G=\frac{\left(m+1\right)A}{2\pi D_{t-r}^{2}}\cos^{m}(\varphi )\cos(\theta )$$

where A is the active photodetector area, D_t−r_ is the transmitter–receiver distance, φ is the irradiance angle, and θ is the incidence angle. This line-of-sight expression is included only to document the conceptual VLC layer proposed for future implementation; it is not used to generate the SUMO traffic-control results reported in this article. A physical implementation will require explicit specification and experimental validation of transmitted optical power, photodetector responsivity and active area, field of view, optical filter and concentrator gains, receiver bandwidth, noise, geometry, occlusion, ambient-light interference, latency, and packet loss.

Ambient illumination can introduce shot noise and receiver saturation. The proposed receiver uses an a-SiC:H/a-Si:H multilayer photodiode with wavelength-selective sensitivity, previously investigated for optical filtering [20]. In the present study, this device property is treated as prior hardware evidence. No warehouse-specific measurements of ambient-light rejection, saturation, signal-to-noise ratio, or bit-error rate are reported, and adaptive-threshold performance is not evaluated experimentally.

#### 2.1.2. Indoor Positioning Based on VLC

**Figure 2:**
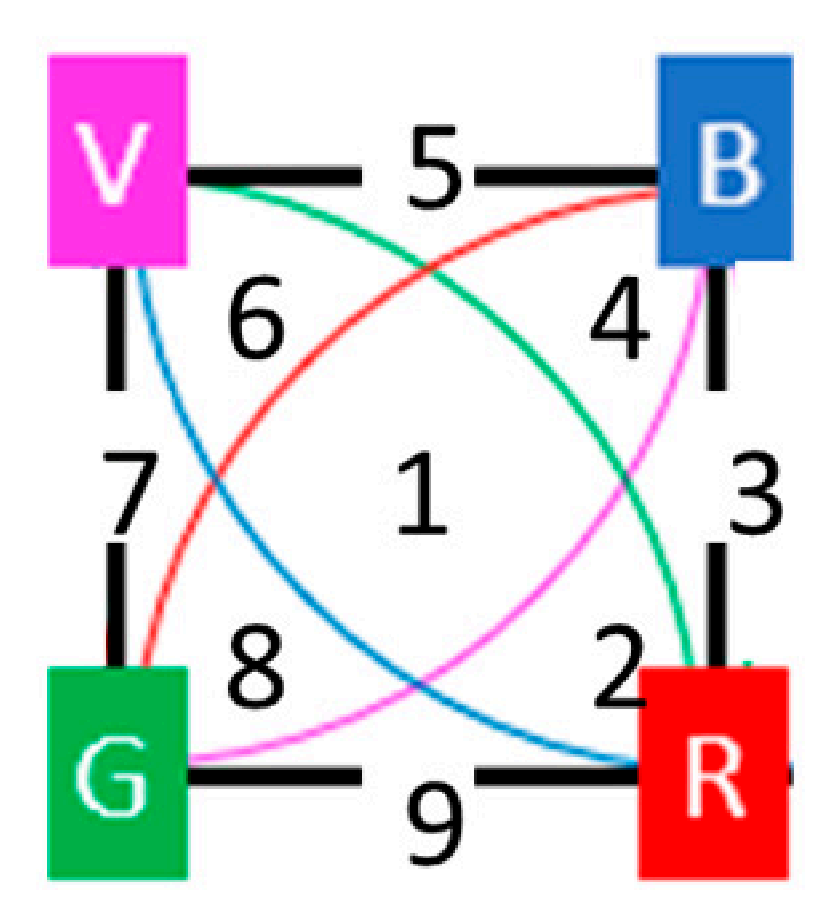
Footprints defined by the square configuration of the VLC transmitter.

The proposed indoor-positioning scheme uses the L2V channel. Four ceiling-mounted emitters arranged in a square generate overlapping optical regions, or footprints, within a navigation cell (Figure 2). The detected combination identifies one of nine regions, while the cell identifier provides the corresponding map location. At the centre of the region depicted in Figure 2, labelled as position 1, all four optical signals transmitted by the modulated LEDs located at the corners of the square are detected. At the corner positions 6, 8, 2, and 4, only three optical signals are detected, corresponding to the Blue–Violet–Green, Violet–Green–Red, Green–Red–Blue, and Red–Blue–Violet combinations, respectively. Along the edges, at positions 5, 7, 9, and 3, the detected optical excitations correspond to the Blue–Violet, Violet–Green, Green–Red, and Red–Blue signal combinations, respectively. Each region is therefore characterised by a unique combination of detected wavelengths. Decoding these signals enables the receiver to identify its position within the optical cell.

Route guidance is then represented through the I2V link. The present study describes this encoding scheme but does not report positioning error, spatial resolution, coverage probability, or warehouse-scale positioning validation.

The positioning frame has a fixed length of 64 bits. The four fields described below occupy 60 bits; the remaining four unassigned bit positions are reserved payload that may be used for future protocol extensions. The currently assigned fields are:

▪ Start of Transmission (SoT): A 4-bit block, 1100, marks the beginning of the positioning frame.
▪ Geolocation (16 bits): An 8-bit cell identifier (reported format XXX0YYY0) and an 8-bit position field identifying the optical footprint.
▪ Message (36 bits): Task-related data, reported to include destination and route information.
▪ End of Transmission (EoT): A 4-bit block, 0011, marks the end of the positioning frame.

#### 2.1.3. VLC Protocols for AGV Communication

Four logical communication modes are defined. L2V provides positioning and downlink guidance; V2V represents the exchange of vehicle state; V2I carries crossing requests from AGVs to the infrastructure; and I2V returns signal-phase or permission information. These message types support the request–response logic used by the controller. Their communication quality is not evaluated in the SUMO experiments.

Line-of-sight blockage is a relevant limitation of VLC in dense warehouses. A hybrid VLC/RF arrangement is proposed as a future resilience mechanism, with VLC as the primary link and RF as a potential fallback during optical blockage. The RF technology, switching policy, handover delay, outage-detection rule, and performance under occlusion are not implemented in the current simulation. The hybrid architecture must therefore be interpreted as a design proposal, not as a demonstrated continuity mechanism.

At each intersection, the agent uses the available traffic state to select one of the permitted signalling phases. The selected action affects the next SUMO state and reward; traffic observations are inputs to the policy, whereas the phase and duration are control outputs.

For each logical communication event, the relevant traffic information is encoded in the fixed 64-bit, mode-dependent structure summarised in Table 1.

Each frame begins with the 5-bit synchronisation word SOF/SYNC = 10,101, followed by an 18-bit time field comprising three 6-bit subfields for hour, minute, and second. A 4-bit ID-start flag (1111) precedes the 4-bit COM field; COM values 1, 2, 3, and 4 identify L2V, V2V, V2I, and I2V messages, respectively. Position is represented by two 4-bit coordinates in y–x order. The named mode-dependent fields occupy the payload region, and all otherwise unassigned bit positions remain reserved payload available for future use. Each frame ends with the 4-bit delimiter EOF = 0000.

Medium access in the proposed deployment would follow an infrastructure-controlled, contention-free TDMA scheme. Each crossing point would act as the coordinator of its optical cell and would broadcast a beacon defining the slot allocation for the following frame. Registered AGVs would transmit V2I and V2V messages only in their assigned uplink slots, while L2V broadcasts and I2V responses would occupy dedicated downlink slots. At most one independent transmitter would be active within each optical interference domain; spatial reuse of a slot would be allowed only between cells with non-overlapping optical footprints, with adjacent crossing-point coordinators exchanging their allocations. Guard intervals would accommodate clock error and transmitter–receiver switching. The 18-bit TIME field represents the message timestamp and is not used as the TDMA slot clock. The 64-bit structures in Table 1 are treated as logical message units carried by the future MAC/PHY layer. Although messages are generated independently by each AGV, access to the shared optical channel would be coordinated through infrastructure-controlled, contention-free TDMA. Upon entering the communication zone, each AGV would be assigned a dedicated uplink time slot, while synchronisation and scheduling information would be broadcast by the infrastructure during a separate I2V downlink interval. Consequently, AGVs sharing the same optical receiver would not transmit simultaneously. This access mechanism was not implemented in the present SUMO experiments, which assume ideal, collision-free logical message delivery.

### 2.2. SUMO Warehouse Traffic Model

The warehouse traffic environment is modelled microscopically in SUMO. Position, speed, lane occupancy, queue length, waiting time, lane-change events, and loading or unloading activity are extracted from the simulator for control and analysis. The VLC elements define the intended information path between vehicles, luminaires, signalling units, and intersection agents, but no physical transmitter, receiver, or channel model modifies the mobility data used by the controller.

#### Layout of the Indoor Environment and AGV Movement

**Figure 3:**
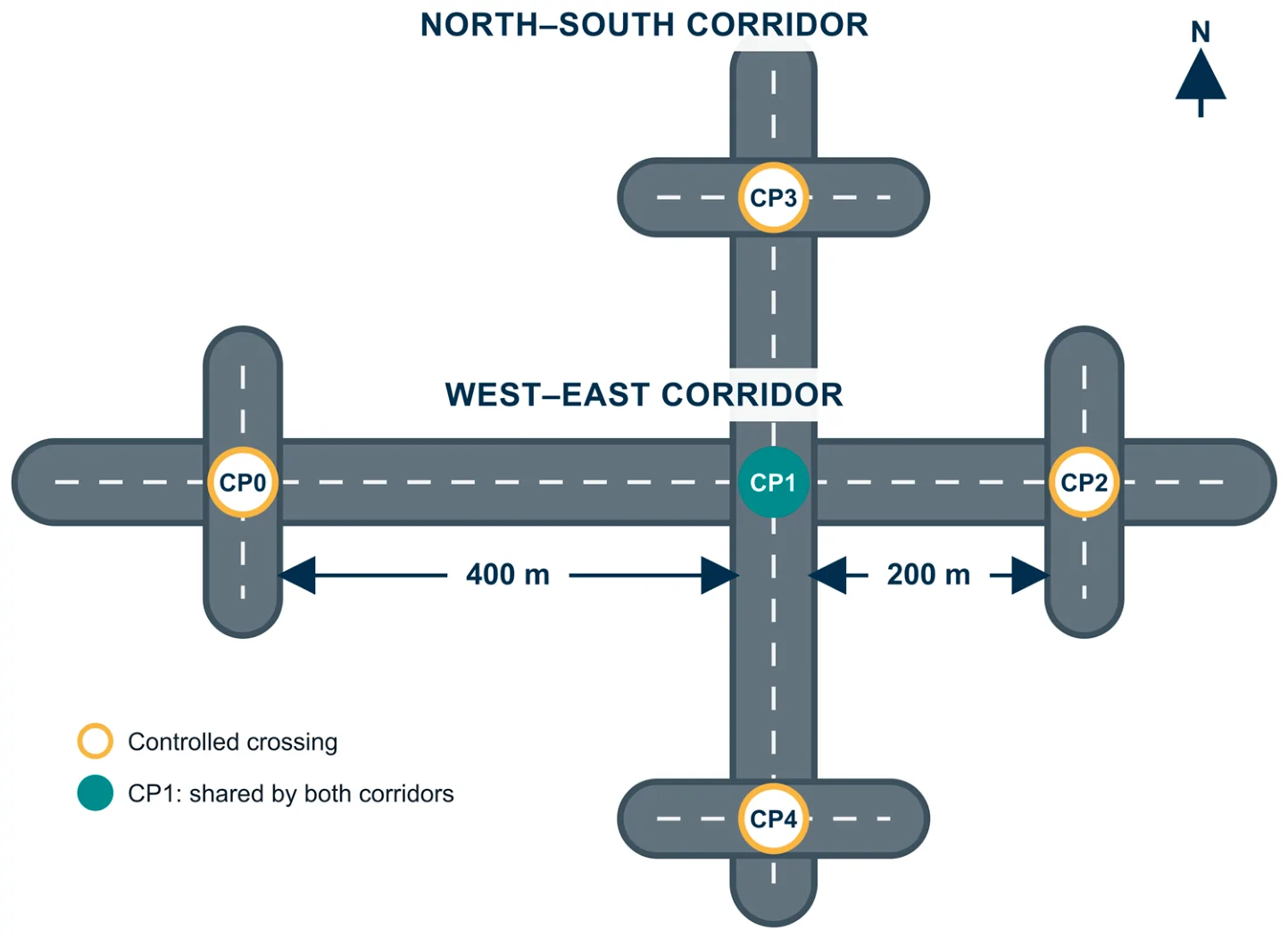
Schematic warehouse layout showing CP0-CP4, the west–east and north–south corridors, the shared crossing CP1, and the asymmetric 400 m and 200 m links.

The simulated warehouse contains five distinct crossing points: CP0, CP1, and CP2 lie on the west–east axis, while CP3, CP1, and CP4 lie on the north–south axis, with CP1 shared by both corridors (Figure 3). CP0–CP1 is 400 m long and CP1–CP2 is 200 m long, creating an asymmetric layout. AGVs originate from the charging or packaging areas and follow predefined SUMO routes through loading and unloading zones. Vehicle arrivals follow a Weibull distribution, and the scenario arrival rates represent the total number of AGVs entering the network per hour. Approximately 65% of this network-wide flow follows CP0–CP1–CP2 and 35% follows CP3–CP1–CP4; approximately 75% of all AGVs proceed straight, and the remaining 25% turn left. SUMO manages lane changing and collision avoidance. The maximum AGV speed is 1.2 m/s (4.32 km/h), each trip contains five to six stops, and each loading or unloading event lasts approximately 32 s. Detailed origin–destination and task-allocation rules remain unavailable.

**Figure 4:**
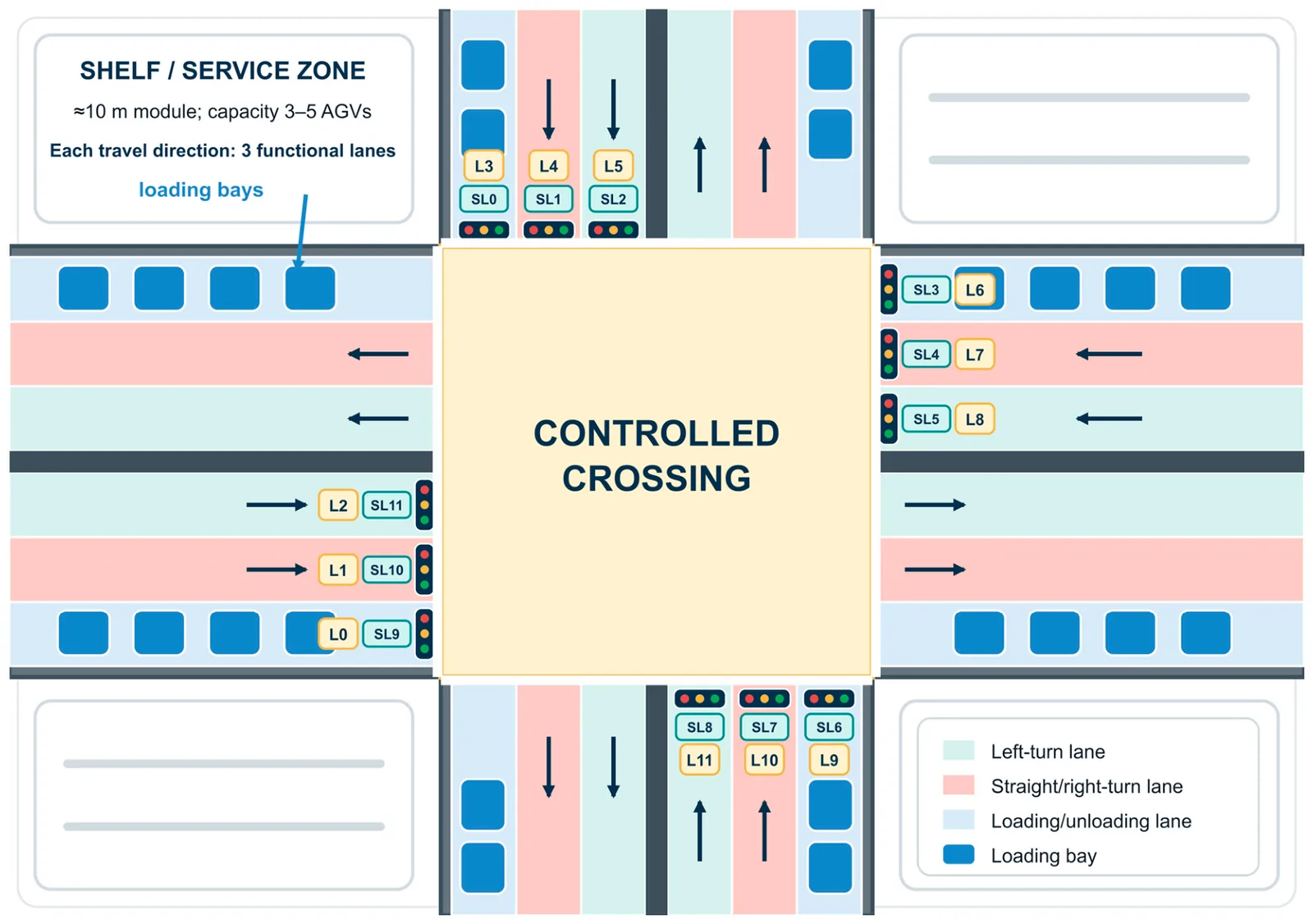
Schematic crossing layout showing the three functional approach lanes, loading/unloading bays, shelf/service zones, stop lines, and signalling units.

Figure 4 provides a detailed view of a crossing point, including its traffic lanes and loading or unloading stops. Each arm has three lanes: one for left turns, one for straight-ahead or right-turn movements, and one for loading and unloading. Shelves approximately 10 m long accommodate three to five AGVs simultaneously before vehicles merge into the traffic lanes.

The arrows correpsond to the direction of the movement along the warehouse. Lanes L0, L3, L6, and L9 are assigned to loading and unloading; L1, L4, L7, and L10 to straight-ahead and right-turn movements; and L2, L5, L8, and L11 to left turns. Twelve signalling lights are defined at each crossing: SL0-SL2 control north-to-south movements, SL3-SL5 east-to-west, SL6-SL8 south-to-north, and SL9-SL11 west-to-east. Within each directional group, the lowest-numbered signal controls the right turn, the intermediate signal the straight-ahead movement, and the highest-numbered signal the left turn. The notation used to describe lanes and signalling lights inside the warehouse are summarized in Table 2.

### 2.3. DQN Intersection Controller

#### 2.3.1. Multi-Agent Adaptive Intersection Control

The learning problem is formulated at intersection level rather than at individual-vehicle level. Each agent controls the signalling phases of one crossing, while AGV motion, route following, lane changing, and service stops remain part of the SUMO environment. Fixed routing and task scheduling are therefore not learned by the controller.

A multi-agent formulation is used because actions at one crossing can alter queues and downstream occupancy at neighbouring crossings. Local control reduces the dimensionality of each decision, while shared-training experience is intended to expose the agents to traffic conditions observed across the warehouse network.

Related warehouse studies have applied MARL to cooperative multi-AGV control [24], energy-efficient routing [25], dynamic scheduling [26], and safety-policy generation under uncertainty [27]. The present study differs by controlling intersection signals rather than vehicle routes or task assignments.

The adopted architecture uses shared parameters and experience during training while retaining local signal control during execution. Specifically, the five agents share one online DQN, one target network, and a common replay memory, consistent with the published base implementation [38]. SUMO, a microscopic road-traffic simulation platform, is adapted to the warehouse setting by representing AGVs as vehicles, warehouse aisles as traffic lanes, and controlled crossing points as signalised intersections. The traffic-control model is otherwise transferred with minimal changes to the warehouse layout and operating conditions.

The scope of the learned policy is restricted to selecting signal phases. Collision avoidance, AGV-level routing, task allocation, and loading-station scheduling are handled by the simulated environment or fixed scenario configuration and are not outcomes of the learned controller.

#### 2.3.2. Distributed DQN Agents: Training and Testing

**Figure 5:**
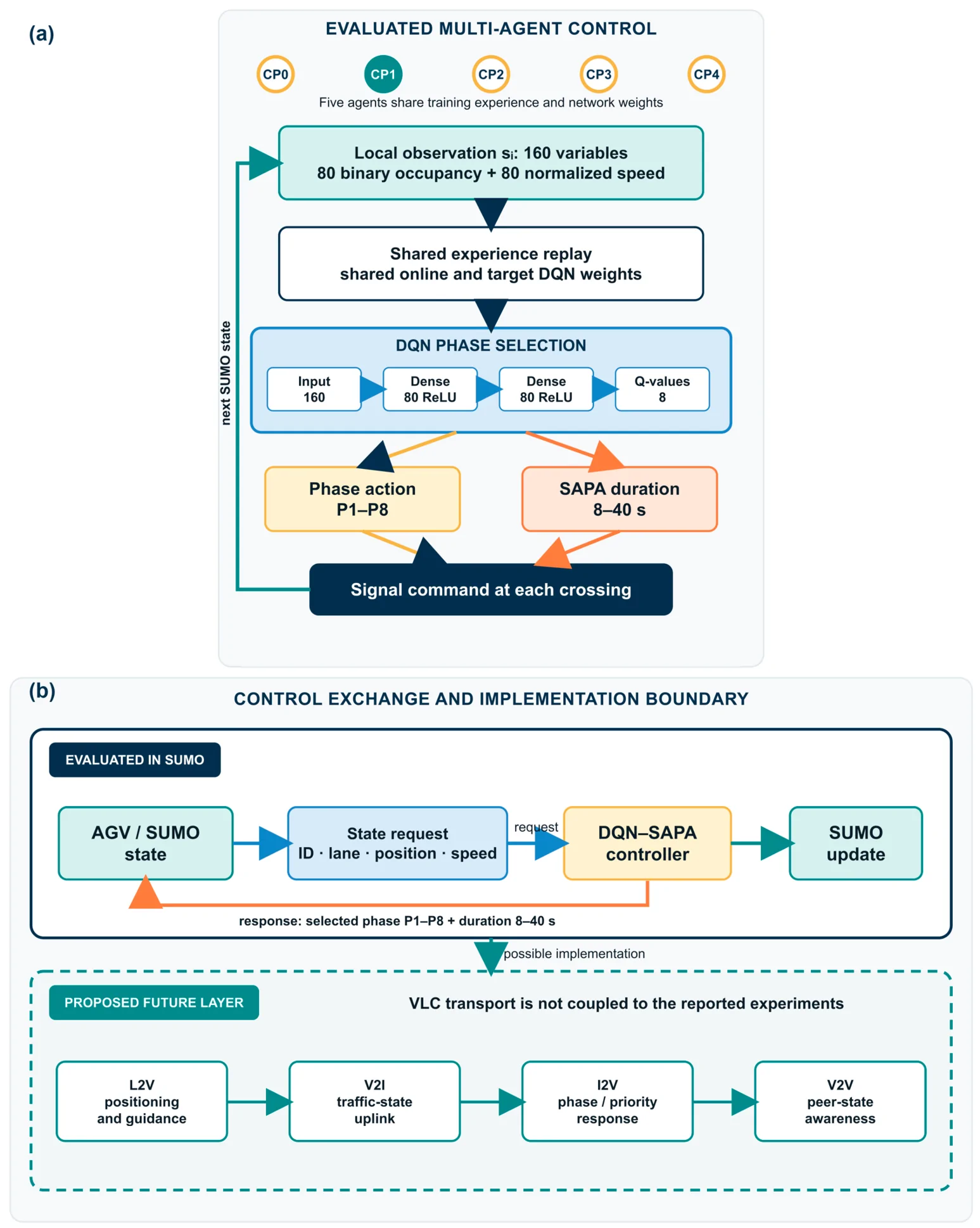
(**a**) Evaluated shared-training DQN architecture, including the 160-variable observation, 80–80 hidden layers, eight phase actions; (**b**) logical SUMO request–response exchange and its separation from the proposed future VLC transport layer.

Each warehouse agent receives a 160-input local state comprising 80 binary cell-occupancy values and 80 normalised cell-speed values for the AGVs approaching its controlled crossing. Figure 5a summarises the resulting shared-training architecture, whereas Figure 5b separates the logical SUMO control exchange from the proposed future VLC transport layer.

As illustrated in Figure 5b, a logical state request provides the controller with the vehicle and mobility information available in SUMO, and the response applies the selected phase. This request–response exchange is represented logically in the simulation. No communication latency, packet loss, collision, or erroneous decoding is included when the controller evaluates the request.

Each DQN approximates the action-value function Q(s, a; θ) with an online network of parameters θ. A separate target network with parameters θ^−^ supplies the reference value used during training. For a non-terminal transition (s_t_, a_t_, r_t_, s_t+1_), the target is

y_t_ = r_t_ + γ max_a_′ Q(s_t+1_, a′; θ^−^), (1)

Here, r_t_ is the immediate reward. The warehouse implementation uses a discount factor γ = 0.75. No special treatment of terminal transitions is applied; each training episode runs for 3600 s, and the target network is updated at the end of the episode. Experiences from all intersection agents enter the common replay memory and update the shared network during centralised training.

The online network is trained by minimising the mean squared temporal-difference error between the target y_t_ and the predicted action value Q(s_t_, a_t_; θ):

L(θ) = E[(y_t_ − Q(s_t_, a_t_; θ))^2^], (2)

The online network uses the inherited mean-squared-error loss and Adam optimiser [38], with a learning rate of 0.0001, a batch size of 128, and γ = 0.75. The confirmed warehouse training comprises one run of 200 episodes of 3600 s each and stops after the 200th episode; the replay memory holds 50,000 samples, and the target network is updated at the end of every episode. Samples are collected during each episode and subsequently used in a training cycle. The previously reported value of 100 epochs is not retained as a confirmed warehouse setting because the exact number of gradient passes per cycle was not specified. Exploration follows a linearly decreasing ε-greedy schedule. With 200 total episodes and n ∈ {1, …, 200} denoting the current episode, the probability of selecting a random exploratory action is ε(n) = 1 − n/200, while the probability of selecting the greedy action is 1 − ε(n) = n/200. With one-based episode numbering, ε(1) = 0.995 and ε(200) = 0; the policy is therefore almost fully exploratory at the beginning of training and fully greedy in the final episode. After the 200th episode, the network from the final episode was retained for evaluation. It was tested in three 6200 s simulations for each reported traffic-demand scenario using seeds 10,000, 10,002, and 10,003; the same seeds were used for the matched fixed-time baseline tests.

The action-to-phase mapping used is: action 0 selects P1 (north–south), action 1 selects P2 (north), action 2 selects P3 (south), action 3 selects P4 (north/south left turns), action 4 selects P5 (west–east), action 5 selects P6 (west), action 6 selects P7 (east), and action 7 selects P8 (west/east left turns). When the controller selects an incompatible movement, the current green is followed by a fixed 4 s yellow interval and then red. Non-selected incompatible movements remain red; their red duration is complementary to the green and yellow intervals assigned to the selected phases and is not an independent DQN action. The warehouse reward is based exclusively on the change in accumulated total waiting time (ATWT) of the AGVs that intend to traverse the controlled intersection:

r_t_ = *atwt_agv_*_,*t*−1_ − *atwt_agv_*_,*t*_, (3)

Waiting for loading-zone service is not included as a separate reward component. A positive reward indicates that the accumulated waiting time of the eligible AGVs has decreased between consecutive decision steps.

Operating regimes are classified after simulation as fluid when the number of AGVs being serviced exceeds the number waiting, at the capacity boundary when the two counts are approximately equal, and saturated when the waiting count is higher. This approach is used to interpret the modelled CP1 scenarios; it does not establish an exact or general warehouse-capacity threshold.

### 2.4. Simulation Configuration, Comparator, and Metrics

Table 3 consolidates the parameters reported for the SUMO environment, VLC architecture and DQN, while distinguishing inherited base-controller settings from warehouse-specific adaptations. The remaining reproducibility items are the detailed origin–destination and task-assignment rules, the demand composition used during DQN training, the online-network gradient-update schedule within each training cycle, ATWT aggregation and boundary handling, the exact SUMO phase-state strings, and the neighbour-aggregation rule.

## 3. Results and Discussion

**Figure 6:**
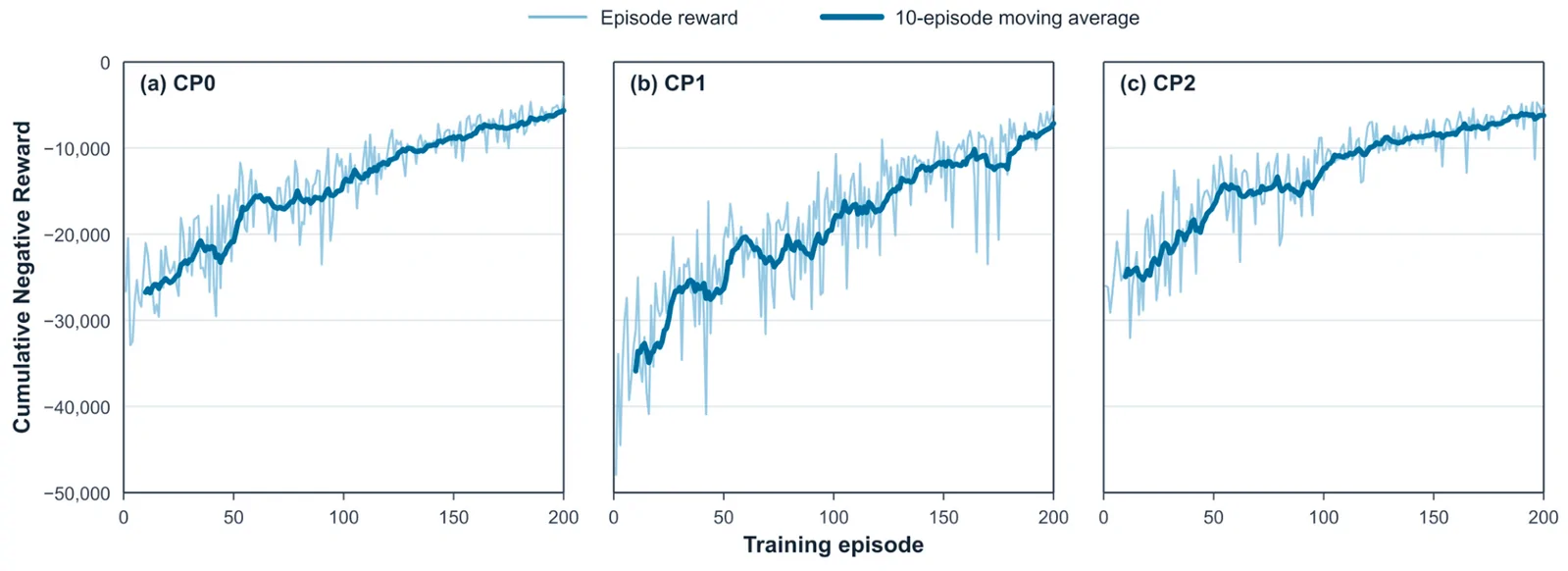
Cumulative negative training reward from the single 200-episode network-training run for the agents at (**a**) CP0, (**b**) CP1, and (**c**) CP2. The light curves show episode-level rewards, and the dark curves show the corresponding 10-episode moving average.

**Figure 7:**
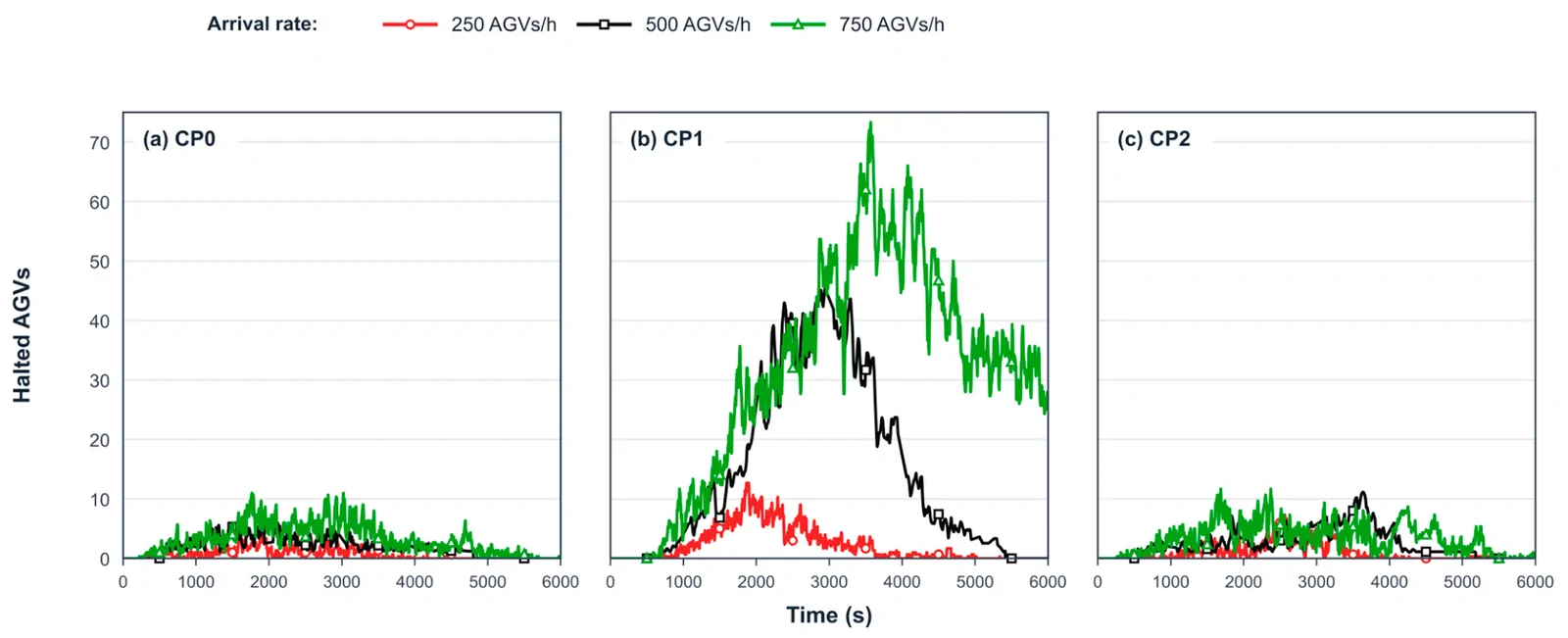
Number of halted AGVs at (**a**) CP0, (**b**) CP1, and (**c**) CP2 along the horizontal axis under the DQN controller for arrival rates of 250, 500, and 750 AGVs/h. Controller curves are arithmetic means of three post-training test simulations.

**Figure 8:**
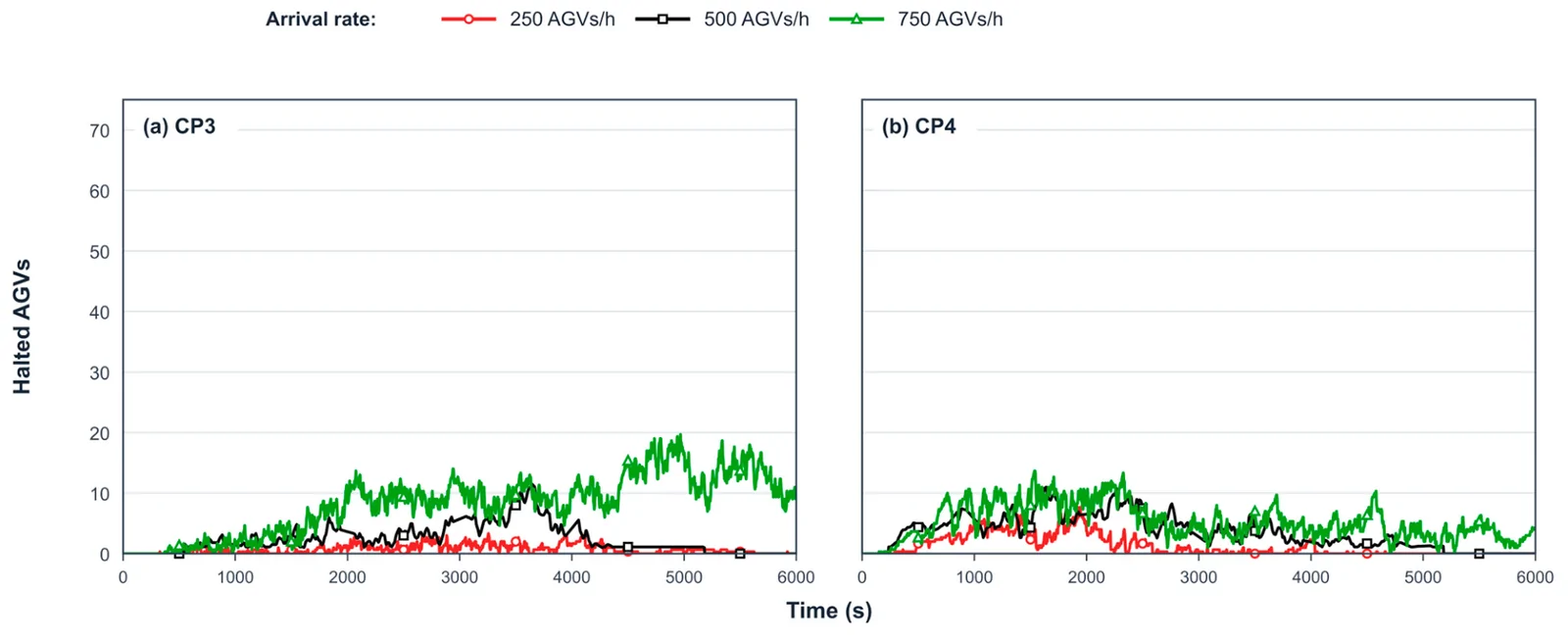
Number of halted AGVs at (**a**) CP3 and (**b**) CP4 along the vertical axis under the controller for arrival rates of 250, 500, and 750 AGVs/h. Each curve is the arithmetic mean of three post-training test simulations.

**Figure 9:**
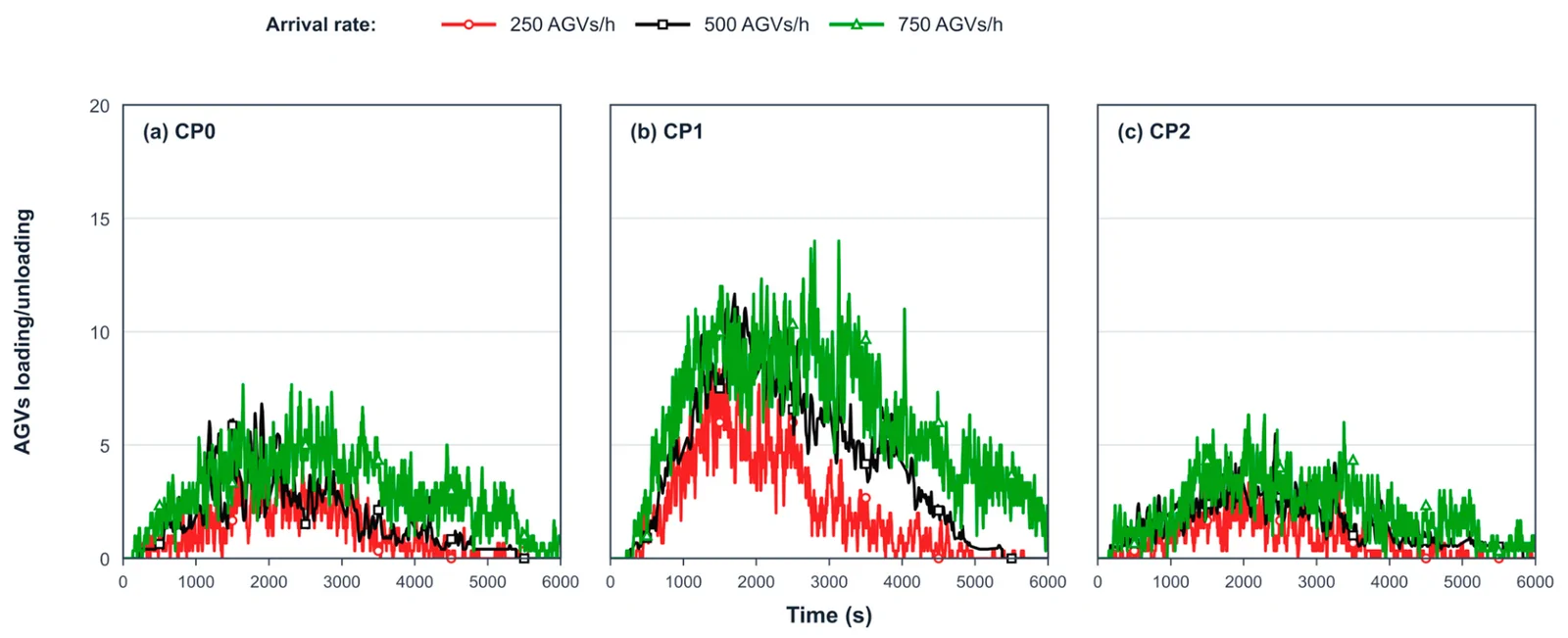
Number of AGVs simultaneously loading or unloading at (**a**) CP0, (**b**) CP1, and (**c**) CP2 along the horizontal axis under the DQN controller for arrival rates of 250, 500, and 750 AGVs/h.

**Figure 10:**
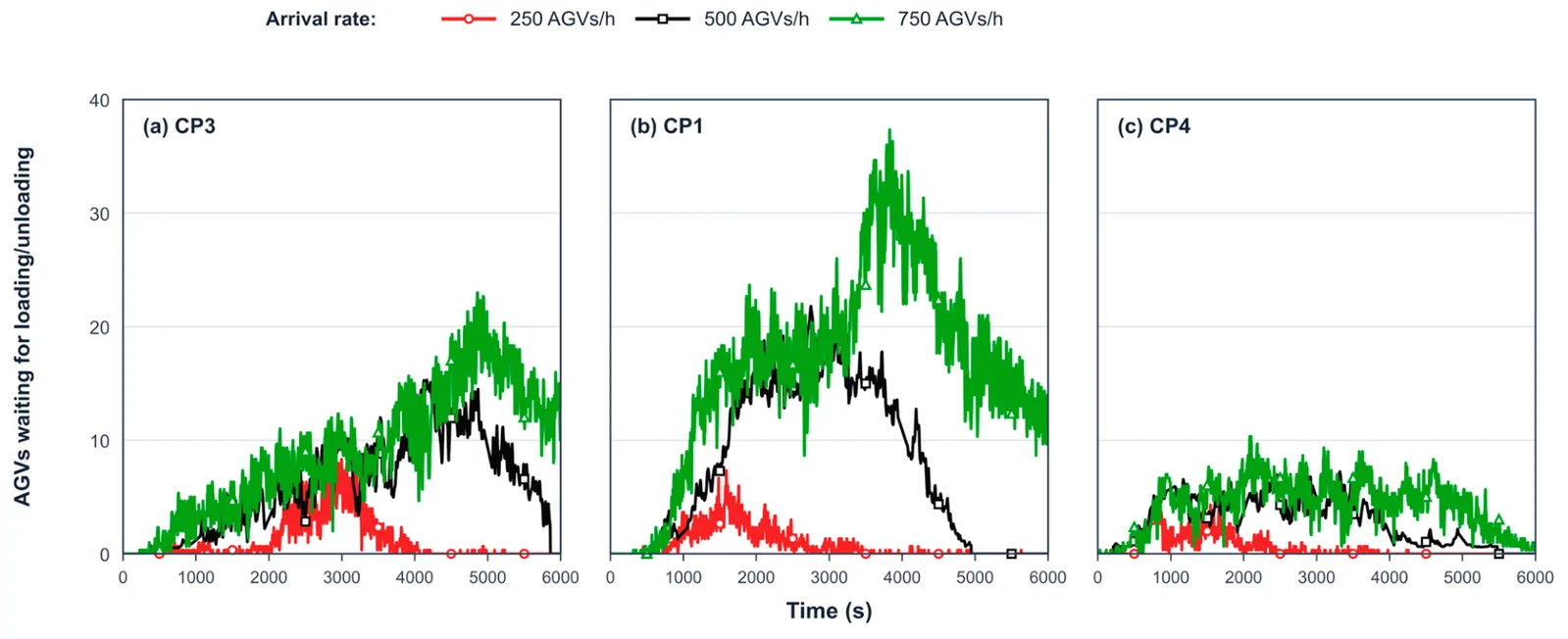
Number of AGVs waiting to access loading or unloading service at (**a**) CP3, (**b**) CP1, and (**c**) CP4 along the vertical axis under the DQN controller for arrival rates of 250, 500, and 750 AGVs/h. Each curve is the arithmetic mean of three post-training test simulations.

**Figure 11:**
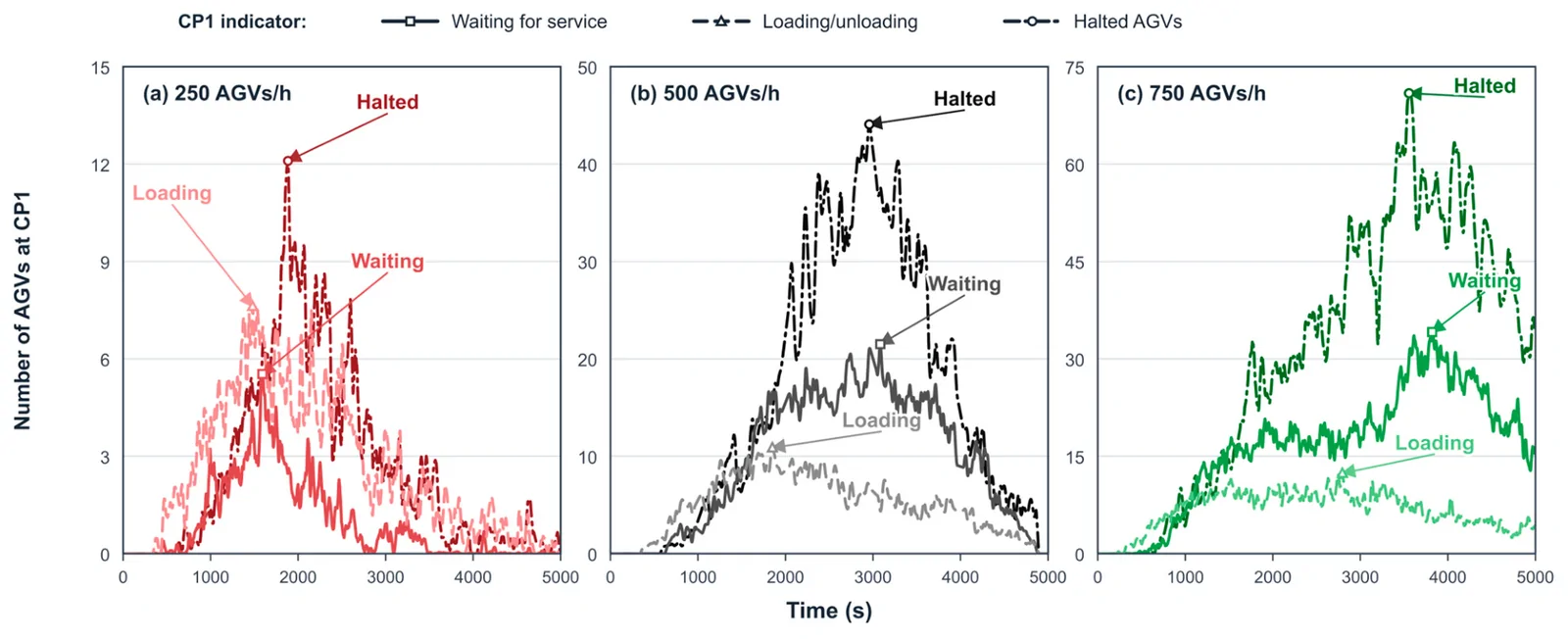
CP1 operating indicators under the DQN controller for arrival rates of (**a**) 250, (**b**) 500, and (**c**) 750 AGVs/h; AGVs waiting to begin service, loading or unloading, and halted.

**Figure 12:**
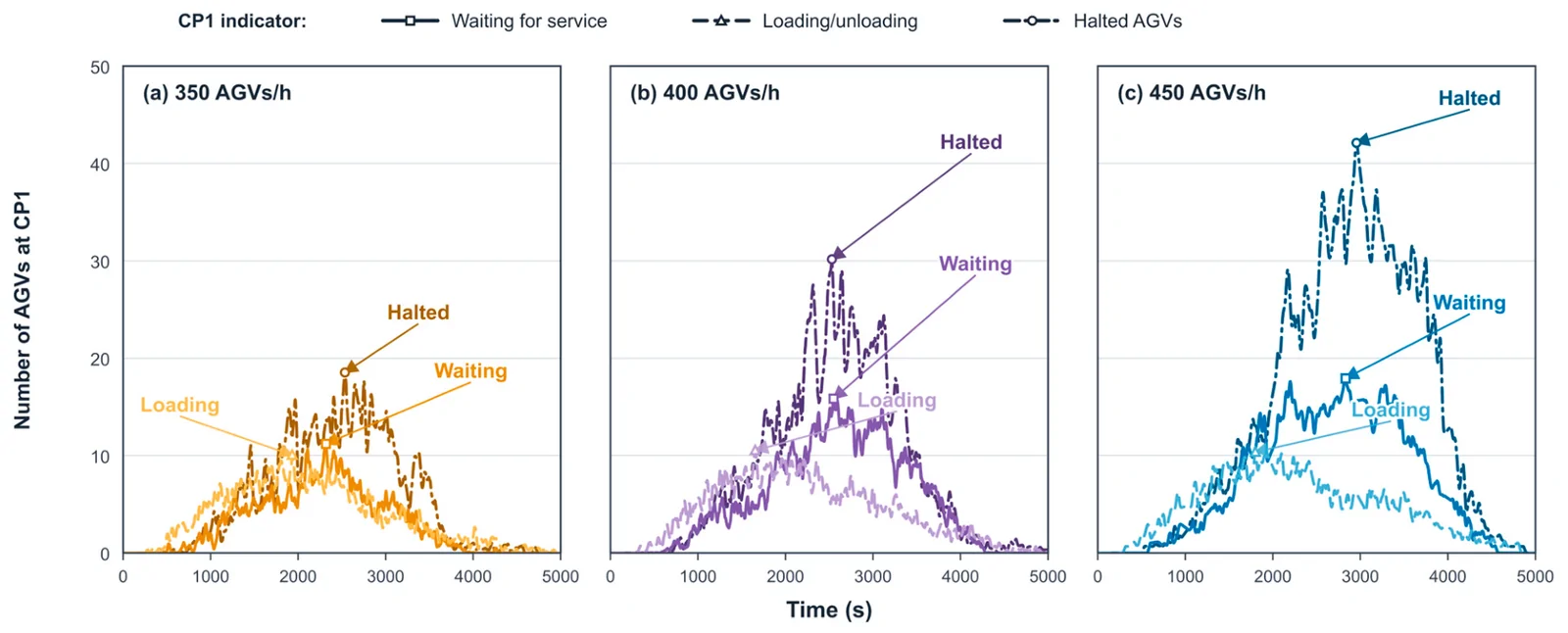
CP1 operating indicators for the refined arrival rates of (**a**) 350, (**b**) 400, and (**c**) 450 AGVs/h; AGVs waiting to begin service, loading or unloading, and halted.

**Figure 13:**
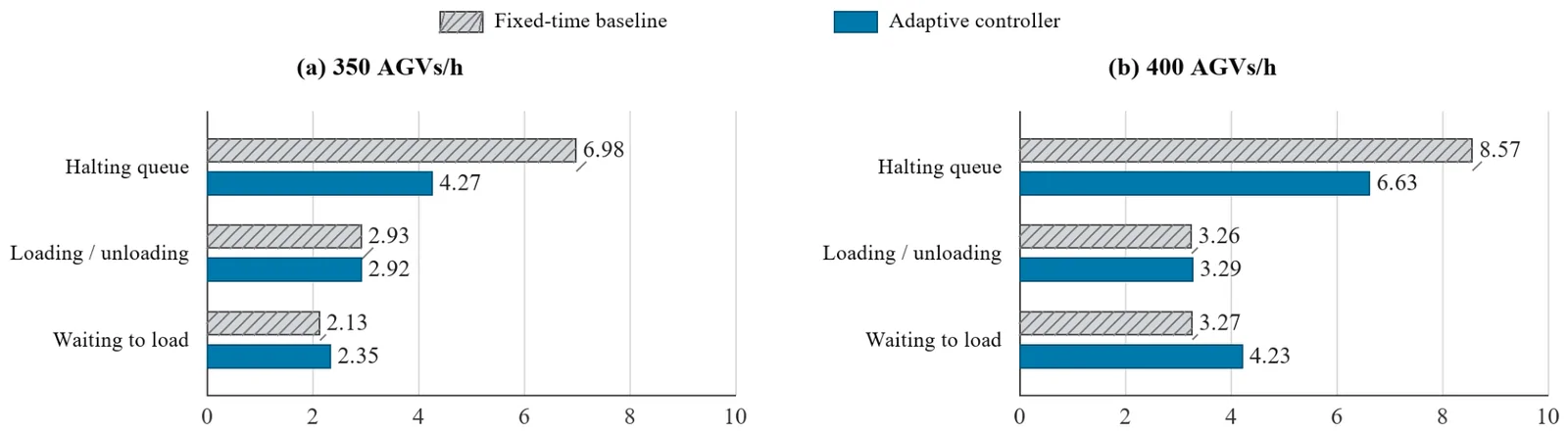
Fixed-time baseline versus adaptive-controller temporal mean numbers of AGVs at CP1 for arrival rates of (**a**) 350 and (**b**) 400 AGVs/h.

**Figure 14:**
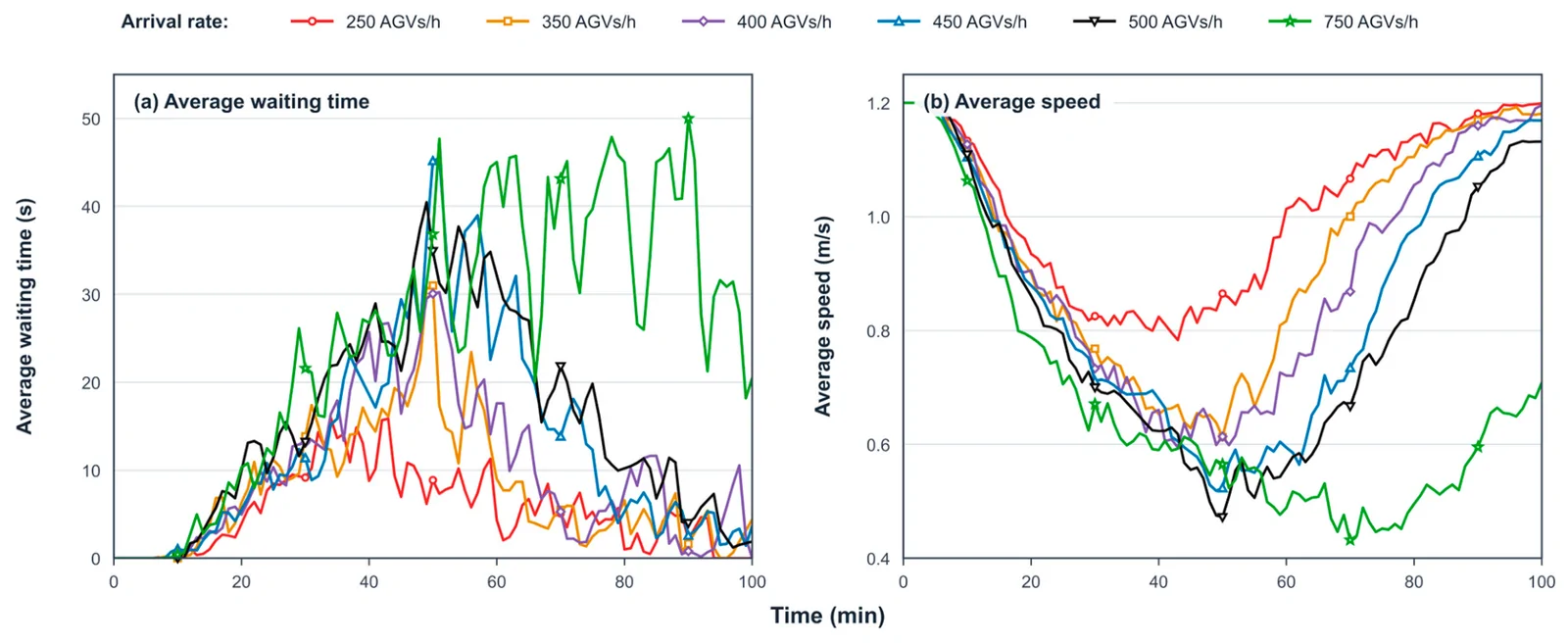
Controller-only traffic-flow performance at CP1 for arrival rates of 250, 350, 400, 450, 500, and 750 AGVs/h: (**a**) average waiting time and (**b**) average speed. Each curve is the arithmetic mean of three post-training test simulations and reports minute-level values without additional moving-average smoothing; no fixed-time baseline is shown.

This section reports simulation results for the DQN controller under the ideal state-availability assumption defined in Section 2. Figure 6 reports the single 200-episode training trajectory. The traffic-result curves in Figure 7, Figure 8, Figure 9, Figure 10, Figure 11, Figure 12, Figure 13 and Figure 14 and the reported controller scenario values are arithmetic means of three post-training test simulations performed with seeds 10,000, 10,002, and 10,003; the same seeds were used for the matched fixed-time tests. The time-series figures use three scenario groups: 250, 500, and 750 AGVs/h for the initial demand sweep; 350, 400, and 450 AGVs/h for refinement around the capacity transition; and the six arrival rates together for the CP1 traffic-flow comparison. For visualisation only, the high-frequency CP1 indicators in Figure 11 and Figure 12 are displayed as centred 30 s moving averages applied to the three-test mean series. This smoothing does not change the temporal means used in the controller–baseline comparison. The individual test series are retained, allowing post-training simulation variability to be quantified; because the network was trained once, this does not quantify variability between independent DQN training runs.

The analysis first examines reward traces and traffic behaviour across the five crossing points, then focuses on CP1, the intersection shared by the west–east and north–south corridors. The reported indicators are vehicles halted at signalling lights, vehicles loading or unloading, vehicles waiting to begin service, average waiting time, and average speed.

Figure 6 presents training rewards. Figure 7, Figure 8, Figure 9, Figure 10, Figure 11, Figure 12 and Figure 14 present controller-only time-series or scenario outputs. Figure 13 is the only figure showing matched fixed-time and adaptive-controller values. The quantitative non-saturated fixed-time comparison is reported in Figure 13 and Table 4 using the 250, 350, and 400 AGVs/h values supplied in the supplementary workbooks; the matched 750 AGVs/h values are reported separately in Section 3.8. Differences are interpreted as controller-level effects under ideal communication and are not attributed independently to VLC.

### 3.1. Training-Reward Analysis

Figure 6 shows the cumulative negative training reward recorded during the single 200-episode training run for the agents controlling CP0, CP1, and CP2. The lighter curves retain episode-to-episode variation, whereas the 10-episode moving average highlights the learning trend without replacing the underlying episode-level rewards. In all three traces, the cumulative training reward becomes progressively less negative, indicating improved performance during this training run.

The CP0 and CP2 traces follow broadly similar ranges, whereas CP1 remains more negative and shows greater short-term variability. This pattern is consistent with CP1 receiving flows from both warehouse axes and containing more loading or unloading activity, although the cause is not isolated experimentally. Figure 6 contains one training trajectory per crossing from the same single network-training run. The 10-episode moving average is a visual trend aid only; the figure therefore demonstrates within-run learning behaviour but does not establish statistical convergence or policy robustness.

### 3.2. Number of Vehicles Waiting in Queues at Signalling Lights in the Horizontal and Vertical Axes

Figure 7 shows the number of AGVs halted at the signalling lights of CP0, CP1, and CP2 for arrival rates of 250, 500, and 750 AGVs/h. The controller curves are arithmetic means of three post-training test simulations.

At CP0 and CP2, the plotted controller queues remain comparatively small and generally below about 10 halted AGVs. CP1 exhibits substantially larger queues, especially at 500 and 750 AGVs/h, consistent with its role as the shared central crossing and with its higher service demand. The 250 AGVs/h case is the least congested of the three; the two higher-demand traces show sustained accumulation at CP1.

Figure 7 is interpreted as a controller-only comparison across demand levels. Matched fixed-time comparisons are presented separately in Figure 13 and Table 4, where numerical baseline values are available for the same scenarios.

Figure 8 shows the corresponding controller queues at CP3 and CP4 on the vertical axis. CP1 is common to both axes and is reported in Figure 7b.

The CP3 and CP4 traces remain below the highest queue levels observed at CP1, including at 750 AGVs/h. This descriptive comparison identifies CP1 as the dominant bottleneck in the modelled layout. The three-test standard deviations reported for the DQN quantify post-training simulation variability, but the small number of tests and absence of independently trained networks do not establish statistical equivalence between the horizontal and vertical axes.

### 3.3. Number of Vehicles Loading/Unloading in Designated Areas of the Horizontal Axis

Figure 9 reports the number of AGVs engaged in loading or unloading at CP0, CP1, and CP2. This indicator represents concurrent service occupancy rather than throughput, because it counts vehicles being serviced at each time step and does not report completed operations per unit time.

CP1 shows the highest concurrent loading or unloading occupancy and approaches the reported capacity of approximately 10 AGVs under the higher-demand scenarios. CP0 and CP2 have lower service occupancy. The plateau at CP1 is consistent with a finite number of service positions, but it should not be interpreted as improved productivity without a corresponding count of completed services or throughput.

### 3.4. Number of Vehicles Waiting in Queues to Begin Loading

Figure 10 shows the queues waiting to access loading or unloading service at CP3, CP1, and CP4. A vehicle enters this queue when its designated service area is occupied or when access to the loading lane is constrained.

At CP1, the 250 AGVs/h case shows relatively little waiting compared with the number in service. Waiting increases markedly at 500 and 750 AGVs/h and exceeds service occupancy for sustained portions of the plotted horizon.

### 3.5. Influence of the AGV Scenario at the Critical Crossing Point

CP1 is the most highly connected crossing in the modelled warehouse and receives traffic from both axes. Figure 11 combines three CP1 indicators, halted AGVs, AGVs loading or unloading, and AGVs waiting to begin service, for arrival rates of 250, 500, and 750 AGVs/h. The centred 30 s moving average is applied to the mean of the three post-training test simulations, reducing short-period fluctuations while retaining the operating trend. Because congestion amplitudes differ markedly, panels (a)–(c) use vertical ranges of 0–15, 0–50, and 0–75 AGVs, respectively; absolute magnitudes should therefore be compared using the axis values rather than the apparent curve heights.

The 250 AGVs/h scenario remains in the least congested operating regime. At 500 and 750 AGVs/h, the waiting-to-load trace exceeds the loading or unloading trace for sustained periods, while the halted-vehicle trace also increases. Under the operating-regime heuristic defined in Section 2.3.2, these patterns are consistent with overload at CP1, but the exact waiting-to-service ratio is not reported.

As the arrival rate increases, concurrent service occupancy at CP1 rises and then plateaus near 10 AGVs, while the waiting and halting traces continue to grow. This divergence indicates that additional arrivals are accumulating upstream after the available service positions become occupied. The plateau reflects the modelled service-capacity constraint; it is not, by itself, evidence of an optimal service level.

The operating-regime classification is based on the relative numbers being serviced and waiting. More vehicles in service than waiting is classified as fluid; approximately equal counts indicate the capacity boundary; and a higher waiting count is classified as saturated. This is a post-processing heuristic for the simulated CP1 layout, not a general warehouse-capacity definition.

### 3.6. Assessment of Vehicle Arrival Limits at CP1

Figure 12 examines intermediate CP1 arrival rates of 350, 400, and 450 AGVs/h using the same three indicators. Each indicator is first represented by its mean across the three post-training test simulations, to which the same centred 30 s moving-average presentation is applied. These scenarios narrow the transition region between the low-demand and overloaded cases shown in Figure 11. In contrast to Figure 11, all three panels use a common vertical scale of 0–50 AGVs, allowing direct visual comparison of the refined scenarios.

The 350 AGVs/h traces are closest to a balance between service and waiting, whereas the 400 and 450 AGVs/h cases show longer intervals in which waiting exceeds service. The figure therefore places the transition to sustained congestion between 350 and 400 AGVs/h for the mean of the three post-training tests and this layout.

Figure 13 presents the controller benchmark for the two refined scenarios, corresponding to 350 and 400 AGVs/h. The comparison uses CP1-specific temporal means rather than the environment-wide means reported in Table 4.

Relative to fixed-time operation, the adaptive controller reduces the mean halting queue at CP1 from 6.98 to 4.27 ± 1.17 AGVs at 350 AGVs/h (38.8%) and from 8.57 to 6.63 ± 0.76 AGVs at 400 AGVs/h (22.6%). Loading/unloading occupancy is essentially unchanged (2.93 versus 2.92 ± 0.07 and 3.26 versus 3.29 ± 0.10 AGVs). Conversely, the mean number waiting to load increases from 2.13 to 2.35 ± 0.19 AGVs at 350 AGVs/h (10.3%) and from 3.27 to 4.23 ± 0.41 AGVs at 400 AGVs/h (29.4%). The standard deviations describe variability across the three post-training DQN simulations. Thus, the controller reduces intersection halting, but the benefit is accompanied by a waiting-to-load trade-off that becomes more pronounced at 400 AGVs/h. These CP1-specific values should not be read as the environment-wide averages summarised in Table 4.

### 3.7. Traffic-Flow Performance

Figure 14 reports minute-level average waiting time and average speed at CP1 for the six controller scenarios from 250 to 750 AGVs/h; no additional moving-average smoothing is applied. The values include motion and the reported 32 s loading or unloading dwell periods, so the plotted average speed is expected to remain below the 1.2 m/s movement limit.

Within the adaptive-controller results, average waiting time increases, and average speed decreases as the arrival rate rises, with the largest deterioration at 500 and 750 AGVs/h. Figure 14 contains no fixed-time curves, so it does not support a controller-versus-baseline speed or travel-efficiency claim. The separately reported 59% improvement concerns average waiting time in the 250, 350, and 400 AGVs/h benchmark scenarios and is quantified in Table 4. The 250 and 350 AGVs/h scenarios have the lowest waiting times and highest average speeds in Figure 14. Performance deteriorates at 400 and 450 AGVs/h and more strongly at 500 and 750 AGVs/h, consistent with the queue and service-capacity patterns in Figure 11 and Figure 12. These within-controller trends identify a layout-specific operating boundary but do not quantify generalisation to other warehouse geometries, service processes, or communication conditions.

Across the three matched scenarios, the unweighted mean of the scenario-specific reductions is 62.9% for the mean signal queue, 59.1% for average waiting time, and 18.0% for AGVs waiting to load. Concurrent loading or unloading occupancy changes by less than 1% in each scenario and shows no systematic improvement. For the DQN, the sample standard deviations across the three post-training tests are 0.23/0.55/0.32 AGVs for the environment-wide signal queue, 1.06/1.84/1.09 s for average waiting time, 0.01/0.02/0.01 AGVs for loading/unloading occupancy, and 0.07/0.23/0.24 AGVs for waiting to load, ordered as 250/350/400 AGVs/h. Between-test variability is reported for the DQN controller; corresponding uncertainty estimates could not be derived for the fixed-time baseline from the available aggregated data. The percentages are descriptive arithmetic means of reductions computed from the aggregated scenario values and are not uncertainty estimates. Because the comparison assumes ideal communication and lacks component ablations, the observed differences are attributable only to the controller condition, not independently to VLC or MARL coordination.

### 3.8. High-Demand Stress Test at 750 AGVs/h

Matched fixed-time and DQN workbooks are available at 750 AGVs/h, allowing this saturated condition to be evaluated separately from the non-saturated benchmark. The environment-wide mean signal queue changes from 10.54 AGVs under fixed-time control to 10.00 ± 1.26 AGVs under DQN, a 5.1% reduction. However, average waiting time increases from 23.63 to 24.97 ± 3.25 s (5.7%), and the mean number of AGVs waiting to load increases from 6.01 to 7.12 ± 0.70 (18.4%). The reported DQN standard deviations are calculated across the three post-training simulations. The small aggregate queue reduction therefore does not represent an overall operational benefit.

The deterioration is concentrated at CP1. Its mean signal queue increases from 13.15 to 30.68 ± 6.67 AGVs (133.3%), concurrent loading/unloading occupancy rises from 4.74 to 5.91 ± 0.08 AGVs (24.7%), and the mean number waiting to load increases from 9.64 to 15.93 ± 1.06 AGVs (65.2%). The DQN standard deviations again refer to the three post-training simulations. These values confirm that DQN loses its advantage at the central bottleneck once the modelled system is saturated.

The degradation at 750 AGVs/h is consistent with a physical discharge-capacity limitation at CP1, which receives traffic from two long approaches of approximately 400 m and can therefore accumulate substantial upstream queues. At this demand level, arrivals exceed the rate at which CP1 can clear the conflicting movements, and the resulting spillback interferes with nearby loading and unloading activity. The controller structure may aggravate this condition: a selected phase is activated for 8 s and is extended only when the DQN selects the same phase again, in further 8 s increments. Under severe saturation, this mechanism may fail to provide sufficiently long and stable service periods for the accumulated queues. Moreover, the reward is based on the accumulated waiting time of AGVs intending to cross the intersection and does not explicitly penalise spillback into loading and unloading areas. The learned policy may therefore improve the immediate traffic state without adequately protecting these service zones. These explanations are consistent with the controller design and observed queues but have not been isolated through causal ablation experiments. Future work will examine an explicit spillback penalty and congestion-dependent phase durations.

### 3.9. Discussion and Limitations

The results identify CP1 as the principal bottleneck and show that the DQN controller reduces queue-related indicators relative to the study-specific fixed-time comparator in the three matched non-saturated scenarios. The largest effects occur in signal queues and average waiting time, whereas the number of AGVs concurrently in service is essentially unchanged. The separate 750 AGVs/h stress test shows that this benefit does not generalise beyond the modelled capacity boundary: environment-wide waiting indicators worsen and the CP1 signal queue more than doubles relative to fixed-time control. The evidence uses one network-training run followed by three post-training simulations per scenario. Dispersion across those three tests characterises stochastic simulation variability after training, but it cannot establish robustness across independently trained networks. The experiments also do not isolate the contributions of DQN and multi-agent coordination. The proposed VLC architecture lies outside the evaluated system, so the reported differences cannot be attributed to the communication medium.

## 4. Conclusions

This study evaluated a DQN-based adaptive intersection controller in an adapted SUMO warehouse layout with five crossing points. The empirical contribution is therefore the assessment of the traffic-control policy under ideal availability of SUMO state variables; no VLC physical or network layer was simulated. Across the reported analyses, CP1, the crossing shared by the west–east and north–south corridors, was the principal bottleneck in the modelled layout.

For the three demand scenarios with matched numerical results (250, 350, and 400 AGVs/h), the controller reduced the mean signal queue by an unweighted average of 62.9% and the average waiting time by 59.1% relative to the study-specific fixed-time comparator. The mean number of AGVs waiting to load decreased by 18.0%, whereas concurrent loading or unloading occupancy changed by less than 1% in each scenario and showed no systematic improvement. Table 4 reports sample standard deviations across the three post-training DQN tests. These percentages are descriptive arithmetic means of the scenario-specific reductions and are not uncertainty estimates or tests of statistical significance.

The results place the transition to sustained congestion at CP1 between 350 and 400 AGVs/h for the mean of the three post-training tests and the reported layout. At 750 AGVs/h, the matched stress test shows a 5.1% reduction in the environment-wide mean signal queue but a 5.7% increase in average waiting time and an 18.4% increase in AGVs waiting to load. At CP1, the mean signal queue rises from 13.15 to 30.68 ± 6.67 AGVs, and the mean number waiting to load rises from 9.64 to 15.93 ± 1.06 AGVs under DQN. The controller advantage therefore does not persist after saturation. This operating boundary is specific to the simulated geometry, service process, demand configuration, and post-processing heuristic and should not be interpreted as a general warehouse saturation threshold.

The observed performance differences must therefore be attributed to the DQN control condition relative to the cyclic fixed-time baseline, not to VLC or localisation. The proposed VLC architecture remains a future implementation concept for conveying position, traffic-state, and control information. Its optical propagation, packet errors, latency, occlusion, receiver saturation, and RF handover have not yet been evaluated in the control loop. The a-SiC:H/a-Si:H photodiode and hybrid VLC/RF elements consequently require future system-level validation before any VLC-enabled operational gain can be claimed.

The findings provide descriptive evidence that DQN intersection control can reduce queue-related indicators relative to the configured fixed-time comparator below saturation, while the 750 AGVs/h stress test defines an important limit to that conclusion. Interpretation remains constrained by the single warehouse layout, inconsistent scenario sets across figures, one network-training run, only three post-training tests per scenario, and incomplete reporting of task assignment and some training procedures. The retained test outputs permit dispersion across the three post-training simulations to be reported, but they do not quantify training variability or replace independent training repetitions. Future work should repeat the complete DQN training process across multiple seeds, increase the test replication count where required, evaluate additional warehouse layouts, compare the method with other adaptive or learning-based controllers under identical scenarios and seeds, and perform DQN and coordination ablations. Controller development should also investigate an explicit loading-zone spillback penalty and phase durations that adapt directly to congestion. A subsequent implementation phase should couple the SUMO control loop to the proposed VLC architecture and quantify the effects of measured packet loss, delay, occlusion, receiver saturation, and VLC/RF handover.

## Figures and Tables

**Table 1:** Mode-dependent 64-bit frame structure for the AGV-VLC protocol.

Mode	SOF/SYNC	Time	ID Flag	COM	Position	Payload	EOF
	5 Bits	18 Bits	4 Bits	4 Bits	8 Bits	Mode-Dependent + Reserved	4 Bits
L2V	10,101	Hour (6) Min (6) Sec (6)	1111	1	y (4) x (4)	Unassigned bits reserved for future use	0000
V2V	10,101	Hour (6) Min (6) Sec (6)	1111	2	y (4) x (4)	Lane (0–7); vehicle no.; Car IDx; Car IDy; no. behind; unused bits reserved	0000
V2I	10,101	Hour (6) Min (6) Sec (6)	1111	3	y (4) x (4)	TL (0–15); vehicle no.; Car IDx; Car IDy; no. behind; unused bits reserved	0000
I2V	10,101	Hour (6) Min (6) Sec (6)	1111	4	y (4) x (4)	TL (0–15); vehicle ID; Car IDx; Car IDy; no. behind; phase; unused bits reserved	0000

**Table 2:** Notation used for warehouse lanes and signalling lights.

Notation	Matrix Notation
Matrix notation	Position coordinates assigned to the ceiling-mounted VLC transmitters: R = red emitter, G = green emitter, B = blue emitter, V = violet emitter
L0, L3, L6, L9	Lanes designated for loading/unloading operations
L1, L4, L7, L10	Lanes accommodating straight and right-turn movements
L2, L5, L8, L11	Lanes reserved for left-turn movements
SL0–SL11	Signalling lights at each intersection - SL0, SL1, SL2: regulate flows north to south - SL3, SL4, SL5: east to west - SL6, SL7, SL8: south to north - SL9, SL10, SL11: west to east

**Table 3:** Reported simulation and controller parameters.

Category	Parameter	Value/Specification
AGV Dynamics	Maximum Speed	1.2 m/s (4.32 km/h)
	Loading/Unloading Time	32 s
	Stops per Trip	5–6 stops
Warehouse Layout	Distance CP0–CP1	400 m
	Distance CP1–CP2	200 m
	Shelf Length	~10 m (capacity: 3–5 AGVs)
	Lanes per Arm	3 (loading, straight/right, left)
Baseline Logic	Cyclic Fixed-Time	Predefined cyclic sequence P1–P8; P1 (north–south) and P5 (west–east): 40 s green; P2, P3, P4, P6, P7, and P8: 20 s green; 4 s yellow after each phase; no separate all-red; total cycle: 232 s
Traffic (SUMO)	Arrival Rates	250, 350, 400, 450, 500, and 750 AGVs/h; total arrivals entering the complete network
	Training and Test Repetitions	Training: 1 × 200 episodes of 3600 s; network from final episode retained. Testing: 3 × 6200 s post-training simulations per scenario, without warm-up; individual outputs retained and controller curves averaged across the three tests
	Mobility Model	SUMO 1.19 microscopic traffic simulation; simulation step: 1 s
	Simulation Seeds	10,000, 10,002, and 10,003 for each scenario; identical seed set used for DQN and fixed-time tests
	Decision/Reward Interval	Variable; equal to the applied signalling-phase duration; next observation, action, and reward calculation occur at phase end
	Route Allocation	Approximately 65% CP0–CP1–CP2 and 35% CP3–CP1–CP4; approximately 75% straight and 25% left turns
	Demand Generation	Weibull-distributed vehicle arrivals
VLC System	LED Wavelengths	620 nm (R), 530 nm (G), 470 nm (B), 405 nm (V)
	Modulation Scheme	On-off keying (OOK)
	Reported Beamwidth	120° (convention to be specified)
	Frame Structure	Fixed 64-bit frame; 18-bit time field (6 bits each for hour, minute, and second); mode-dependent fields; unassigned bit positions reserved as payload
MARL/DQN	State Space	160 inputs: 80 binary occupancy values +80 normalised speed values; no pedestrian inputs
	Reward	Change in accumulated waiting time of AGVs intending to traverse the controlled intersection; pedestrian component removed; loading-zone waiting not included as a separate term
	Neural Network Architecture	Shared online/target DQN; 2 hidden layers (80 ReLU units each)
	Activation Function	ReLU
	Action Space	8 discrete phases P1–P8; pedestrian phase P9 removed
	Adaptive Signal Timing	Green: 8–40 s in 8 s increments; yellow: 4 s; red: complementary
	DQN Training Settings	Adam/MSE inherited from [38]; lr = 0.0001; batch = 128; γ = 0.75; replay memory = 50,000 samples; 200 × 3600 s episodes; random-action ε(n) = 1 − n/200 for n = 1, …, 200 (ε(1) = 0.995; ε(200) = 0); target network updated at episode end

**Table 4:** Descriptive environment-wide fixed-time versus DQN comparison for 250, 350, and 400 AGVs/h. Fixed-time entries are reported as means; DQN entries are mean ± sample standard deviation across the three post-training simulations with seeds 10,000, 10,002, and 10,003. Values are ordered by scenario; relative reduction is calculated as 100 × (B − C)/B, where B is the fixed-time value and C is the DQN mean.

Metric	Fixed-Time Mean	DQN Mean ± SD	Reduction (%) by Scenario; Mean
Mean signal queue (AGVs)	2.99/5.24/6.45	1.08 ± 0.23/1.88 ± 0.55/2.53 ± 0.32	63.8%/64.1%/60.9%; 62.9%
Average waiting time (s)	14.09/19.04/21.82	5.29 ± 1.06/7.79 ± 1.84/9.64 ± 1.09	62.4%/59.1%/55.8%; 59.1%
Mean AGV loading/unloading	1.08/1.52/1.72	1.08 ± 0.01/1.51 ± 0.02/1.72 ± 0.01	0.7%/0.7%/−0.4%; no systematic change
Mean AGVs waiting to load	0.77/1.82/2.55	0.58 ± 0.07/1.51 ± 0.23/2.22 ± 0.24	24.4%/17.0%/12.7%; 18.0%

## Data Availability

The data supporting the findings of this study are available from the corresponding author upon reasonable request.

## Abbreviations

The following abbreviations are used in this manuscript:

AGV	Automated Guided Vehicle
ATWT	Accumulated Total Waiting Time
COM	Communication link
CP	Crossing Point
CTDE	Centralised Training with Decentralised Execution
DQN	Deep Q-Network
DRL	Deep Reinforcement Learning
EOF	End of Frame
EoT	End of Transmission
ID	Identification of the AGV
I2V	Infrastructure-to-Vehicle
LED	Light Emitting Diode
LoS	Line of Sight
L2V	Lamp to Vehicle
MAC	Medium Access Control
MARL	Multi-Agent Reinforcement Learning
MUX	Multiplexed signal
MSE	Mean Squared Error
OOK	On-Off-Keying
PHY	Physical Layer
RGBV	Red Green Blue Violet
RF	Radio Frequency
RL	Reinforcement Learning
SOF	Start of Frame
SoT	Start of Transmission
SUMO	Simulation of Urban Mobility
SYNC	Synchronisation
VLC	Visible Light Communication
SL	Signalling Light
TDMA	Time Division Multiple Access
V2I	Vehicle to Infrastructure
V2V	Vehicle to Vehicle
V2X	Vehicle to Everything
